# *Rubus phantasmaticus* (Rosaceae), an overlooked new species of *R.* subg. *Malachobatus* from southwestern China

**DOI:** 10.3897/phytokeys.281.204346

**Published:** 2026-10-27

**Authors:** Qiu-Ping Wang, Xiao-Yong Dai, Feng Yang, Ting-Ting Wang, Qing Zhou, Huan-Chong Wang

**Affiliations:** 1 School of Ecology and Environmental Science, Yunnan University, Kunming 650091, Yunnan, China School of Ecology and Environmental Science, Yunnan University Kunming China; 2 Guizhou Academy of Forestry, Guiyang 550005, Guizhou, China Guizhou Academy of Forestry Guiyang China; 3 Yunnan Hubo Environmental Protection Science and Technology Co., Ltd., Kunming 650091, Yunnan, China Yunnan Hubo Environmental Protection Science and Technology Co., Ltd. Kunming China; 4 Herbarium of Yunnan University, Kunming 650091, Yunnan, China Herbarium of Yunnan University Kunming China

**Keywords:** Biogeography, cryptic diversity, molecular phylogenetics, new species, Rubeae

## Abstract

Rubus subg. Malachobatus is morphologically well-circumscribed at the subgeneric level, but its infrasubgeneric relationships remain unclear, and many taxa have been subject to significant taxonomic disputes. Based on molecular phylogenetic analyses of nuclear ribosomal ITS and complete plastome datasets, combined with comprehensive morphological comparisons, a new species from southwestern China is described here as *R. phantasmaticus* Q. P. Wang, X. Y. Dai & Huan C. Wang. Both datasets consistently resolve *R. phantasmaticus* as nested within R. subg. Malachobatus and most closely related to *R. hypopitys*. Morphologically, *R. phantasmaticus* clearly differs from *R. hypopitys* in its growth habit, leaf shape, and inflorescence architecture. Additionally, the two species are geographically adjacent but exhibit non-overlapping distributions. *Rubus phantasmaticus* has long been overlooked and was recently treated as a variety of *R. chrysobotrys*, but both morphological comparisons and molecular phylogenetic evidence indicate that *R. phantasmaticus* represents a distinct taxon with no close phylogenetic affinity to *R. chrysobotrys*.

## Introduction

*Rubus* L. (Rosaceae) is a cosmopolitan and species-rich genus comprising approximately 700 species worldwide (Thuan 1968; Robertson 1974; Yu and Lu 1985; Lu and Boufford 2003). The genus is widely distributed across all continents except Antarctica (Focke 1910, 1911, 1914; Alice and Campbell 1999; Alice et al. 2015), with the highest species richness occurring in East Asia, Europe, and North America (Bailey 1945; Thuan 1968; Robertson 1974; Lu 1983; Yu and Lu 1985; Lu and Boufford 2003). *Rubus* exhibits remarkable morphological diversity, ranging from herbaceous perennials to deciduous or evergreen shrubs. Its stems may be erect, climbing, arching, or creeping, and its leaves can be simple, palmately compound, or pinnately compound (Yu and Lu 1985; Lu and Boufford 2003; Clark et al. 2007; Clark and Finn 2008). *Rubus* represents a globally renowned resource of wild fruit plants, with the aggregate fruits of nearly all species being edible. Cultivated representatives, including blackberry and raspberry, are economically important fruit crops worldwide (Howarth et al. 1997; Alice and Campbell 1999). *Rubus* species are adapted to a wide range of environments, occurring from arctic to tropical regions, from lowlands to high elevations, on acidic to alkaline soils, under arid to humid climates, and in habitats ranging from deeply shaded understories to fully exposed sites (Clark and Finn 2011; Ibarra-Morales et al. 2013). Their diverse growth forms and broad ecological amplitude make *Rubus* species valuable for ecological restoration, vegetation reconstruction, and soil and water conservation (McDowell 2002; Zasada and Tappeiner 2003).

*Rubus* represents a well-defined natural group, characterized by distinct morphological features that readily distinguish it from allied genera, and there has been little controversy regarding its generic boundaries (Alice and Campbell 1999; Morden et al. 2003; Yang and Pak 2006; Yang et al. 2012; Wang et al. 2016; Carter et al. 2019). Its monophyly has been consistently corroborated by numerous molecular phylogenetic studies (Wang et al. 2016; Carter et al. 2019; Okada et al. 2020; Huang et al. 2023; Gao et al. 2023). At the species level, however, *Rubus* is widely recognized as a notoriously difficult group (Robertson 1974; Lu 1983; Richards et al. 1996). The genus comprises a large number of species with a broad geographic distribution, and the prevalence of apomixis, interspecific hybridization, and polyploidy gives rise to exceptionally complex morphological variation (Sudre 1908–1913; Gustafsson 1943; Yu and Lu 1985; Alice et al. 2001), rendering species delimitation particularly challenging (Bammi and Olmo 1966; Mimura et al. 2014; Šarhanová et al. 2017; Carter et al. 2019). Since *Rubus* was established by Linnaeus (1753), extensive taxonomic research has been conducted on this group by botanists worldwide (Focke 1868, 1874, 1910, 1911, 1914; Sudre 1908–1913; Koidzumi 1913; Nakai 1918; Bailey 1941–1945; Heslop-Harrison 1968; Yu and Lu 1985; Naruhashi 2001), providing an essential foundation for understanding the diversity of this taxonomically challenging group. Notably, Focke’s monograph Species Ruborum (Focke 1910, 1911, 1914) remains the only comprehensive worldwide treatment of *Rubus* to date. In his monograph, Focke recognized over 450 species and classified them into 12 distinct subgenera. In Focke’s classification, three subgenera, namely R. subg. Malachobatus (Focke) Fritsch, R. subg. Idaeobatus (Focke) Focke, and R. subg. Rubus (= subg. *Eubatus* Focke), collectively account for approximately 80% of the species in the genus. With the rapid development of molecular systematics, the infrageneric phylogenetic relationships of *Rubus* have been substantially clarified. Multiple molecular phylogenetic studies have demonstrated that, among the subgenera recognized by Focke, only R. subg. Anoplobatus (Focke) Focke, R. subg. Orobatus Focke, and R. subg. Rubus are monophyletic (Alice and Campbell 1999; Carter et al. 2019), whereas the remaining subgenera are non-monophyletic (Alice and Campbell 1999; Morden et al. 2003; Yang and Pak 2006; Alice et al. 2008; Yang et al. 2012; Wang et al. 2016; Okada et al. 2020). Building upon Focke’s classification and integrating mainly complete plastid genome data, Huang et al. (2023) proposed a new subgeneric classification, dividing the genus into 10 subgenera and thereby largely resolving the non-monophyly of most subgenera originally circumscribed by Focke.

Rubus subg. Malachobatus, one of the most species-rich groups within the genus, is predominantly distributed in Asia, with only a few species extending southward into Australia and the Pacific Islands (Kalkman 1987; Bean 1997; Thompson 1997). This subgenus is typically distinguished from other subgenera by its scandent shrubby habit, simple leaves, stipules free from the petiole, and aggregate fruits adnate to a receptacle that remains solid at maturity (Yu and Lu 1985; Lu and Boufford 2003). It is characterized by substantial morphological variability, and species boundaries among closely related taxa are often poorly defined, making species delimitation difficult when based solely on morphological characters. Furthermore, most species in this subgenus are polyploids, many of which are allopolyploids (Bammi 1965; Thompson and Zhao 1993; Thompson 1995; Naruhashi et al. 2002; Michael 2006; Wang et al. 2015), reflecting an exceptionally complex evolutionary history (Thompson 1997; Thompson and Zhao 1993; Wang et al. 2019). Previous phylogenetic studies have demonstrated that R. subg. Malachobatus, as circumscribed by Focke, is not monophyletic (Alice and Campbell 1999; Alice et al. 2008; Carter et al. 2019; Huang et al. 2023). Under the revised circumscription proposed by Huang et al. (2023), R. sec. Lineati Focke was excluded because of its phylogenetically isolated position, whereas R. subg. Dalibardastrum Focke, which was resolved as nested within R. subg. Malachobatus, was merged into the latter, thereby rendering R. subg. Malachobatus a well-supported monophyletic group. Although the circumscription of R. subg. Malachobatus is now largely resolved, its internal classification remains poorly understood. Owing to limited taxon sampling, insufficient molecular variation, and a complex evolutionary history, the interspecific relationships, species limits, and phylogenetic framework of this subgenus remain unresolved, making it one of the most taxonomically difficult groups within *Rubus*.

As currently circumscribed, Rubus subg. Malachobatus comprises approximately 120–130 species, more than 100 of which occur in China, representing roughly 80% of the species diversity of the subgenus and making China a major center of distribution and diversity for this group. In recent years, with intensified field surveys and the increasing application of molecular systematic approaches to species delimitation, a substantial number of new taxa within this subgenus have been described (Wang et al. 2016, 2017; Xiong et al. 2019, 2025; Chen et al. 2024a, 2024b; Bi and Lin 2024). During field investigations and herbarium studies of *Rubus* in China, an unusual taxon belonging to R. subg. Malachobatus was encountered that did not morphologically match any known species documented in Yu and Lu (1985) or Lu and Boufford (2003). This taxon is herein described as *R. phantasmaticus* Q. P. Wang, X. Y. Dai & Huan C. Wang. Further examination revealed that this taxon corresponded to R. chrysobotrys var. glandulosus L.T.Lu, recently published by Bi and Lin (2024). Its placement within *R. chrysobotrys* Hand.-Mazz. therefore required reassessment through morphological comparisons and molecular phylogenetic analyses. To clarify its phylogenetic position and taxonomic status, molecular phylogenetic analyses were conducted using ITS and complete plastome datasets, together with comprehensive morphological comparisons with its close relatives.

## Materials and methods

### Morphological analysis

A comprehensive morphological comparison of *Rubus phantasmaticus*, *R. hypopitys* Focke, *R. chrysobotrys*, and other closely related species was conducted by integrating field investigations with examinations of herbarium specimens from YUKU and digital specimens or images from online databases, including JSTOR Global Plants (http://plants.jstor.org/), the Chinese Virtual Herbarium (http://www.cvh.ac.cn/), the Global Biodiversity Information Facility (http://www.gbif.org), and the Plant Photo Bank of China (http://ppbc.iplant.cn/). Field observations of wild populations and detailed morphological examinations were carried out. The key characters assessed included plant habit, indumentum on all plant parts, leaf blade shape, shape of the leaf base and apex, leaf division pattern, size and division of stipules and bracts, petiole length, calyx shape and division, petal color, number of stamens and pistils, and fruit color.

### Taxon sampling, DNA extraction, sequencing, assembly, and annotation

A total of 41 taxa were sampled for this study, including five outgroup species. Sequence data for most species were obtained from GenBank, while the remaining 12 species were collected in the field, with voucher specimens deposited in the Herbarium of Yunnan University (YUKU). Following previous phylogenetic studies (Xiang et al. 2016; Zhang et al. 2017), species from three closely related genera were selected as outgroups: *Geum* L. (*G. japonicum* Thunb. and *G. aleppicum* Jacq.), *Fallugia* Endl. (*F. paradoxa* (D.Don ex Tilloch & R.Taylor) Endl. ex Torr.), and *Rosa* L. (*R. odorata* (Andrews) Sweet and *R. chinensis* Jacq.). Detailed information for all samples is provided in Table 1, and all sequences used in this study are available in GenBank.

**Table 1.** Species information and GenBank accession numbers for all sequences.

**Taxon**	**GB accession numbers of ITS**	**GB accession numbers of cpDNA**	**Voucher specimens of ITS**	**Voucher specimens of cpDNA**
*Rubus alceifolius*	PZ520299*	PZ524856*	LS17603(YUKU)	LS17603(YUKU)
*Rubus buergeri*	PZ520300*	PZ524857*	LM26279(YUKU)	LM26279(YUKU)
*Rubus chrysobotrys*	PZ520307*	PZ524859*	GS20645(YUKU)	GS20645(YUKU)
*Rubus calycacanthus*	PZ520296*	PZ524848*	XY10768(YUKU)	XY10768(YUKU)
*Rubus gongshanensis*	PZ520298*	PZ524855*	LS20280(YUKU)	LS20280(YUKU)
*Rubus gongshanensis*	PZ520297*	PZ524854*	LS20339(YUKU)	LS20339(YUKU)
*Rubus hypopitys*	PZ520306*	PZ524860*	FM16941(YUKU)	FM16941(YUKU)
*Rubus lyi* (*Rubus faberi*)	PZ520304*	PZ524851*	SJ3942(YUKU)	SJ3942(YUKU)
*Rubus malifolius*	N/A	PZ524853*	N/A	MLP30835(YUKU)
*Rubus phantasmaticus*	PZ520305*	PZ524849*	WS30518(YUKU)	WS30518(YUKU)
*Rubus rufus*	PZ520302*	N/A	GS20674(YUKU)	GS20674(YUKU)
*Rubus rufus*	N/A	PZ524852*	SJ3112(YUKU)	SJ3112(YUKU)
*Rubus rufus* var. *palmatifidus*	PZ520301*	PZ524858*	LC21702(YUKU)	LC21702(YUKU)
*Rubus setchuenensis*	PZ520303*	PZ524850*	TQ10269(YUKU)	TQ10269(YUKU)
*Rubus idaeus*	AF055757	OR972717	N/A	N/A
*Rubus amphidasys*	AY083367	N/A	N/A	N/A
*Rubus swinhoei*	EF034143	NC_077571	N/A	N/A
*Rubus treutleri*	EF034146	OP747376	lice, Sutherland, & Dorji 0409 (WKU)	
*Rubus parviflorus*	EF050804	OR972719	N/A	N/A
*Fallugia paradoxa*	FPU90805	KY419999	N/A	N/A
*Rubus alceifolius*	KP093155	N/A	N/A	N/A
*Rubus acuminatus* var. *puberulus*	KU881053	N/A	N/A	N/A
*Rubus alceifolius*	KU881054	N/A	N/A	N/A
*Rubus amphidasys*	KU881058	N/A	N/A	N/A
*Rubus feddei*	KU881087	N/A	N/A	N/A
*Rubus hypopitys* var. *hanmiensis*	KU881094	N/A	N/A	N/A
*Rubus lasiotrichos*	KU881117	OP747362	N/A	N/A
*Rubus pinnatisepalus*	KU881149	N/A	N/A	N/A
*Rubus setchuenensis*	KU881167	MW238765	N/A	N/A
*Rubus stipulosus*	KU881173	OP747374	N/A	N/A
*Rubus chamaemorus*	LC624368	OP747409	N/A	W.C. Wang 91-40(PE)
*Rubus buergeri*	LC624396	N/A	N/A	N/A
*Rosa odorata*	MG584527	PP191137	N/A	N/A
*Geum japonicum*	MH809015	NC_077561	N/A	N/A
*Rosa chinensis*	MH809041	NC_065867	N/A	N/A
*Geum aleppicum*	MT881459	NC_060733	N/A	N/A
*Rubus argutus*	N/A	OR972714	N/A	N/A
*Rubus chrysobotrys*	N/A	OP747391	N/A	N/A
*Rubus chrysobotrys*	N/A	PP566884	N/A	N/A
*Rubus gongshanensis*	N/A	OP747394	N/A	Qinghai-Tibet Team 8398(PE)
*Rubus incanus*	N/A	MT274119	N/A	N/A
*Rubus longisepalus*	N/A	NC_059904	N/A	N/A
*Rubus menglaensis*	N/A	OP747403	N/A	N/A
*Rubus occidentalis*	N/A	NC_060646	N/A	N/A
*Rubus occidentalis*	N/A	OR972718	N/A	N/A
*Rubus parviaraliifolius*	N/A	MT274122	N/A	N/A
*Rubus poliophyllus*	N/A	OP747407	N/A	Z.Z.Li 307(PE)
*Rubus refractus*	N/A	OP747411	N/A	N/A
*Rubus taiwanicola*	N/A	NC_057631	N/A	N/A
*Rubus takesimensis*	N/A	NC_037991	N/A	N/A
*Rubus trifidus*	N/A	NC_046585	N/A	N/A
*Rubus treutleri*	PP464083	N/A	N/A	N/A
*Rubus buergeri*	PQ214987	N/A	N/A	N/A
*Rubus ellipticus*	PQ214994	NC_056929	N/A	N/A
*Rubus setchuenensis*	PQ215003	N/A	N/A	N/A
*Rubus swinhoei*	PQ215008	N/A	N/A	N/A

*Represents new sequencing data.

Fresh leaf material was collected in the field and dried in silica gel for subsequent analyses. Total genomic DNA was extracted from silica-gel-dried leaf tissue using a modified cetyltrimethylammonium bromide method (mCTAB; Ivanova et al. 2008), followed by purification with the Tiangen Plant Genomic DNA Kit (DP305). DNA quality was assessed using a NanoDrop 1000 spectrophotometer and verified by 0.8% agarose gel electrophoresis. Paired-end libraries were constructed using the TruSeq DNA Sample Preparation Kit (Illumina, San Diego, CA, USA) and sequenced on the Illumina NovaSeq 6000 platform, with a read length of 150 bp and an insert size of approximately 350 bp. Raw sequencing data, approximately 3 Gb per sample, were filtered using Trimmomatic v. 0.39 (Bolger et al. 2014) to remove low-quality reads and adapter contamination, and read quality was assessed with FastQC v. 0.11.9 (http://www.bioinformatics.babraham.ac.uk/projects/fastqc/). On average, approximately 2 Gb of clean data were obtained per library for plastome assembly and annotation. Sequencing was performed by Novogene Co., Ltd. (Tianjin, China).

*De novo* assembly of the nuclear ribosomal DNA (nrDNA) and complete plastomes was performed using genome-skimming data in GetOrganelle v. 1.7.5 (Jin et al. 2020) with default parameters. Plastome annotation was performed using CPGAVAS2 (Shi et al. 2019) and GeSeq (Tillich et al. 2017), with the plastome of *Rubus irenaeus* (GenBank accession number: PP566888) as a reference. Annotation results were manually inspected and corrected in Geneious Prime v. 2025.0.2 (Kearse et al. 2012). Chloroplot (Zheng et al. 2020; https://irscope.shinyapps.io/chloroplot/), a comprehensive and interactive tool for visualizing organelle genomes, was used to generate the final circular map of the plastome.

### Phylogenetic analyses

Phylogenetic analyses were conducted separately using the complete plastome dataset and the ITS dataset. The plastome and ITS datasets included 42 and 39 sequences, respectively. Species names and GenBank accession numbers for these sequences are listed in Table 1. The plastome and ITS sequences were assembled into separate matrices using Geneious Prime v. 2025.0.2 (Kearse et al. 2012). Sequence alignment was performed using MAFFT v. 7.505 (Katoh and Standley 2013) with the --auto strategy and default parameters. The resulting alignments were subsequently trimmed and optimized using trimAl v. 1.2 (Capella-Gutiérrez et al. 2009). The best-fit nucleotide substitution models were selected using ModelFinder v. 2.2.0 (Kalyaanamoorthy et al. 2017) based on the Bayesian information criterion (BIC). Maximum likelihood (ML) analyses were conducted using IQ-TREE v. 2.2.0 (Nguyen et al. 2015; Minh et al. 2020) under the best-fit models, with 5,000 ultrafast bootstrap (UFBoot) replicates (Minh et al. 2013). Bayesian inference (BI) analyses were performed using MrBayes v. 3.2.7a (Ronquist et al. 2012). Two independent Markov chain Monte Carlo (MCMC) runs were conducted for 5,000,000 generations, with sampling every 1,000 generations. The initial 25% of the samples were discarded as burn-in. All phylogenetic analyses were performed within PhyloSuite v. 2 (Zhao et al. 2025). The resulting phylogenetic trees were visualized and edited using the online tool tvBOT (Xie et al. 2023).

## Results

### Plastome characteristics and gene content of *Rubus phantasmaticus* and its relatives

**Figure 1.**
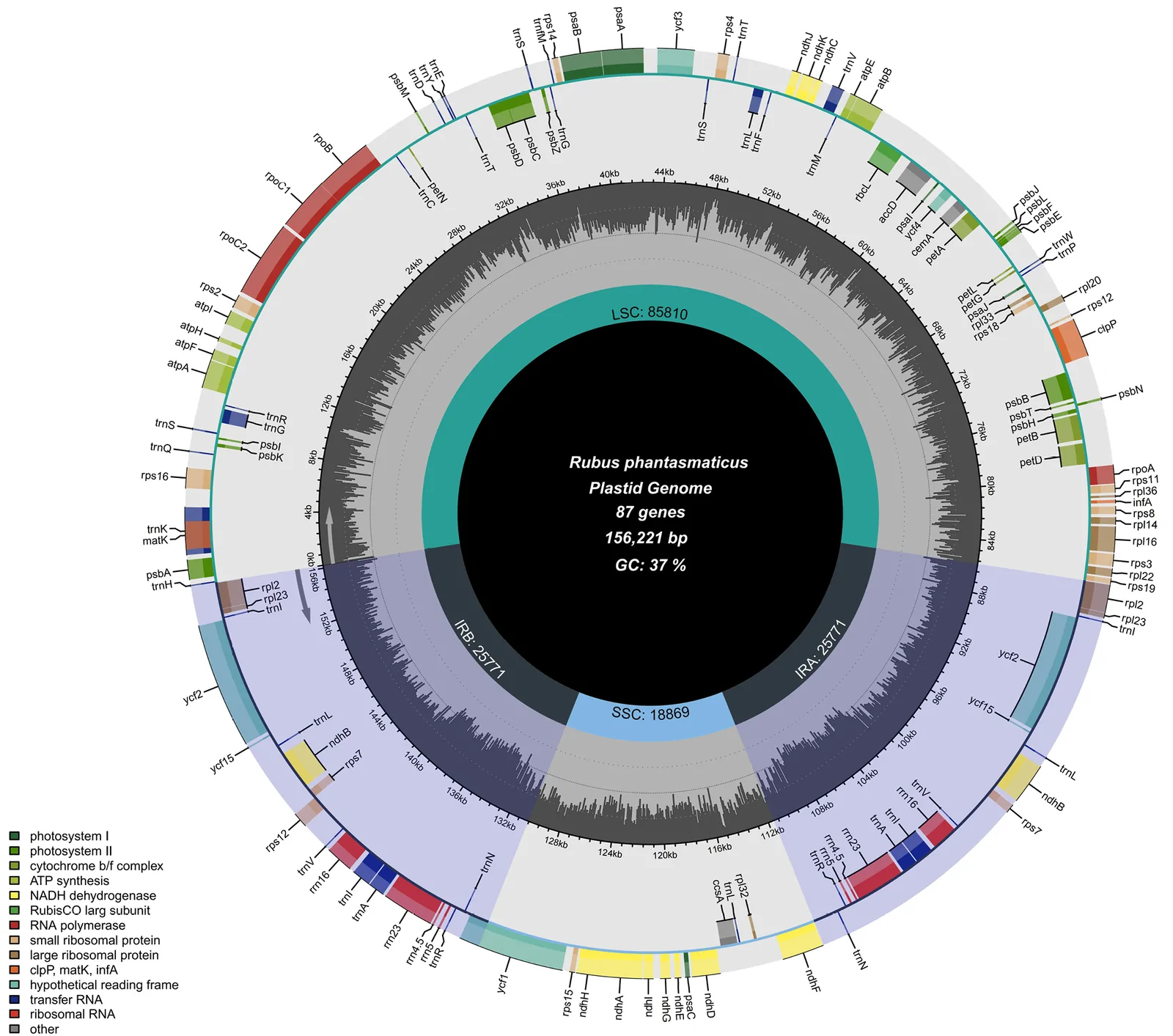
Circular chloroplast genome map of *Rubus phantasmaticus*. The circular genome map illustrates the large single-copy (LSC) region, the small single-copy (SSC) region, and the pair of inverted repeats (IRa and IRb). Functional categories are distinguished by different colors, as shown in the legend. Genes placed along the outer ring are oriented in the counterclockwise direction, while those located on the inner ring are oriented clockwise. The innermost gray histogram represents fluctuations in GC content throughout the genome.

The complete plastomes of *Rubus phantasmaticus*, *R. hypopitys*, and *R. chrysobotrys* were sequenced and analyzed. All three plastomes exhibit the typical circular quadripartite structure, comprising a large single-copy region (LSC), a small single-copy region (SSC), and a pair of inverted repeats (IRs) (Fig. 1). The total plastome lengths range from 156,221 bp in *R. phantasmaticus* to 156,304 bp in *R. chrysobotrys*, with that of *R. hypopitys* being 156,232 bp. The LSC regions range from 85,810 bp in *R. phantasmaticus* to 85,885 bp in *R. chrysobotrys*, with that of *R. hypopitys* being 85,828 bp. The SSC regions range from 18,869 bp in *R. phantasmaticus* to 18,879 bp in *R. chrysobotrys*, with that of *R. hypopitys* being 18,876 bp. The length of each IR region ranges from 25,764 bp in *R. hypopitys* to 25,771 bp in *R. phantasmaticus*, with that of *R. chrysobotrys* being 25,770 bp. The overall GC content of the plastomes is highly conserved at 37.2% across the three species. Among the three major genomic regions, the IR regions show the highest GC content, 42.8% in all three species, followed by the LSC regions, 35.1% in all three species, whereas the SSC regions show the lowest GC content, 31.2% in *R. hypopitys* and *R. phantasmaticus* and 31.1% in *R. chrysobotrys*. Each plastome encodes 132 genes, including 87 protein-coding genes, 37 tRNA genes, and eight rRNA genes. Further synteny analyses indicate that gene order and structural features are highly conserved and consistent with those of other reported *Rubus* plastomes. No significant gene losses, inversions, or rearrangements were detected.

### Phylogenetic analyses

**Figure 2.**
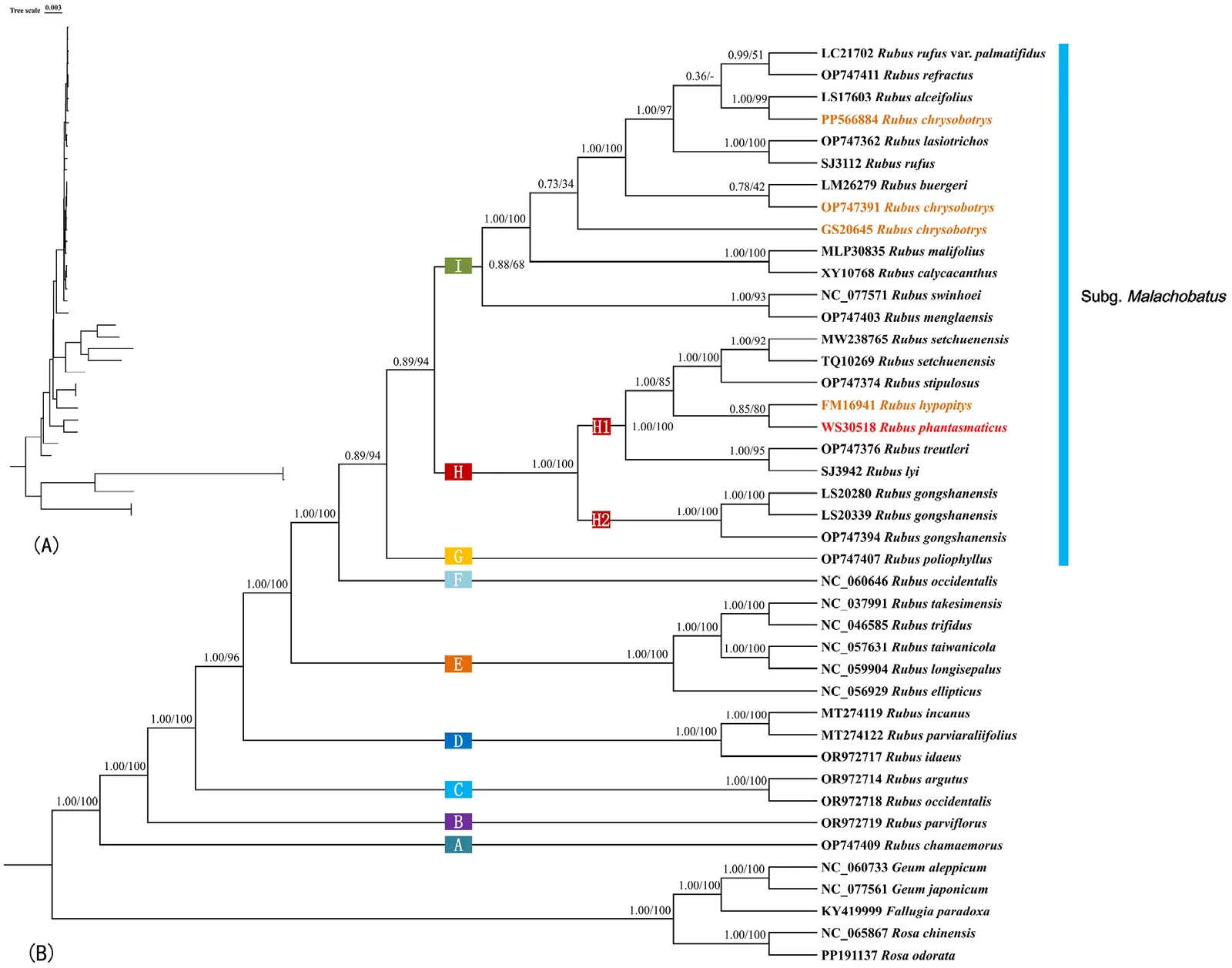
Phylogenetic tree of *Rubus* based on the complete plastome. **A**. Phylogram of *Rubus*; **B**. Cladogram of *Rubus*. Maximum likelihood (ML) bootstrap support (BP) values and Bayesian inference (BI) posterior probabilities (PP) are shown on the branches. *R. phantasmaticus* is highlighted in red. Major lineages are labeled as Clades A–I.

The complete plastome dataset comprised 42 sequences, including 37 ingroup accessions representing 33 taxa of *Rubus* and five outgroup accessions from Geum, *Fallugia*, and *Rosa*. Among these, 12 plastomes were newly sequenced and assembled in this study. After alignment and trimming, the final matrix was 151,743 base pairs (bp) in length and included 9,986 parsimony-informative sites, 3,978 singleton sites, and 137,779 constant sites. Phylogenetic analyses were performed using maximum likelihood (ML) and Bayesian inference (BI). ML analysis was conducted in IQ-TREE (Nguyen et al. 2015; Minh et al. 2020), and BI analysis was carried out in MrBayes v. 3.2.7a (Ronquist et al. 2012). The best-fit substitution model for each analysis was selected using ModelFinder (Kalyaanamoorthy et al. 2017) under the Bayesian information criterion (BIC), yielding GTR+F+R4 for the ML analysis and GTR+F+I+G4 for the BI analysis. The plastome-based phylogenetic tree recovered *Rubus* as a strongly supported monophyletic group (Fig. 2; PP = 1.00, BS = 100). Within *Rubus*, nine major clades (Clades A–I) were resolved, most of which received strong support (BS ≥ 95 and PP ≥ 0.95). The phylogenetic results placed *R. phantasmaticus* within the strongly supported Clade H (PP = 1.00, BS = 100), which belongs to R. subg. Malachobatus. Within this clade, *R. phantasmaticus* formed a moderately supported subclade with *R. hypopitys* (PP = 0.85, BS = 80). This subclade was sister to another subclade comprising two accessions of *R. setchuenensis* Bureau & Franch. and one accession of *R. stipulosus* T.T.Yu & L.T.Lu (PP = 1.00, BS = 100), and together these four species formed a well-supported subclade (PP = 1.00, BS = 85). This subclade was sister to the clade formed by *R. treutleri* Hook.f. and *R. lyi* H.Lév. (PP = 1.00, BS = 95), whereas the three accessions of *R. gongshanensis* T.T.Yu & L.T.Lu constituted a well-supported subclade (PP = 1.00, BS = 100) sister to all remaining members of Clade H.

**Figure 3.**
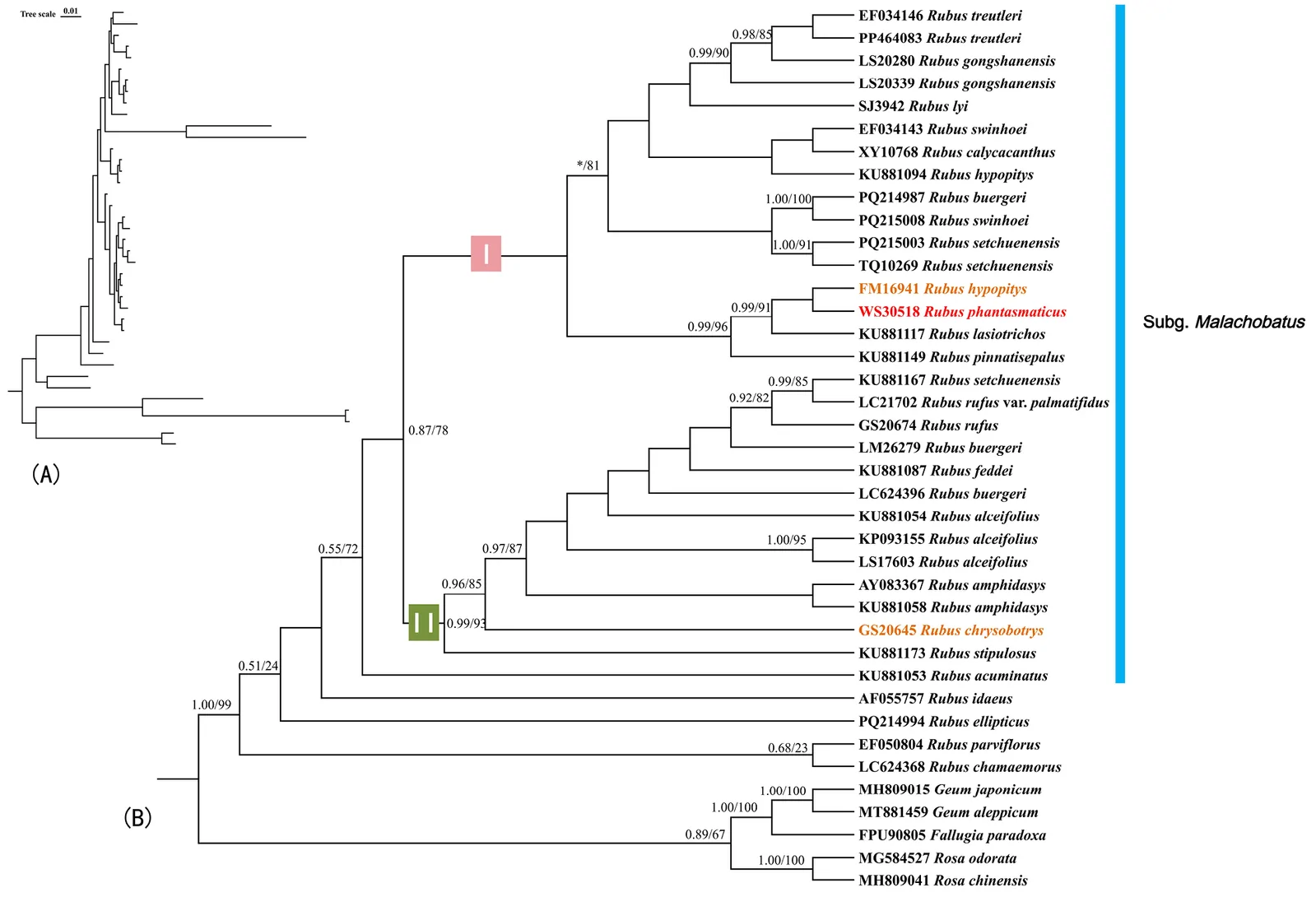
Phylogenetic tree of *Rubus* based on nuclear ribosomal ITS sequences. **A**. Phylogram of *Rubus*; **B**. Cladogram of *Rubus*. Maximum likelihood (ML) bootstrap support (BP) values and Bayesian inference (BI) posterior probabilities (PP) are shown on the branches. *R. phantasmaticus* is highlighted in red. Major lineages are labeled as Clades I–II.

The ITS dataset comprised 39 sequences, including 34 ingroup accessions representing 26 *Rubus* taxa and five outgroup accessions from *Geum*, *Fallugia*, and *Rosa*. After alignment and trimming, the final ITS matrix consisted of 618 bp, including 152 parsimony-informative sites, 68 singleton sites, and 398 constant sites. The best-fit nucleotide substitution models selected under the BIC were TNe+G4 for the ML analysis and K2P+G4 for the BI analysis. The ITS-based phylogenetic tree recovered *Rubus* as a strongly supported monophyletic group (Fig. 3; PP = 1.00, BS = 99). ITS phylogenetic analyses placed *Rubus phantasmaticus* within Clade I. *R. phantasmaticus* was most closely related to *R. hypopitys*, *R. lasiotrichos* Focke, and *R. pinnatisepalus* Hemsl., and together these four species constituted a well-supported subclade (PP = 0.99, BS = 96). The remaining species in Clade I included *R. treutleri*, *R. gongshanensis*, *R. lyi*, *R. swinhoei* Hance, *R. calycacanthus* H.Lév., *R. buergeri*, and *R. setchuenensis*. In contrast, *R. chrysobotrys* was nested within Clade II, indicating a more distant phylogenetic relationship to *R. phantasmaticus*.

### Taxonomic treatment

#### Rubus phantasmaticus

Taxon classificationPlantaeRosalesRosaceae

Q.P.Wang, X.Y.Dai & Huan C.Wang sp. nov.

11450C92-6996-59BC-B6A1-091B90B217CC

urn:lsid:ipni.org:names:77461686-1

Figs 4, 5

= Rubus chrysobotrys var. glandulosus L.T.Lu, syn. nov. Type: China. Sichuan Province, Leibo County, Qinghe Village, 24 August 1989, *Q. S. Zhao 1608* (holotype: PE [barcode 01790026!]).

##### Type.

China. Yunnan Province: Wenshan County, Bozhu Village, 23°21'37.19"N, 103°51'33.34"E, alt. ca. 2,281 m, 23 July 2025, *Q. P. Wang et al. WS30518* (holotype: YUKU [barcode 02074899!]; isotypes: YUKU [barcode 02074900!, 02074901!, 02074902!]).

**Figure 4.**
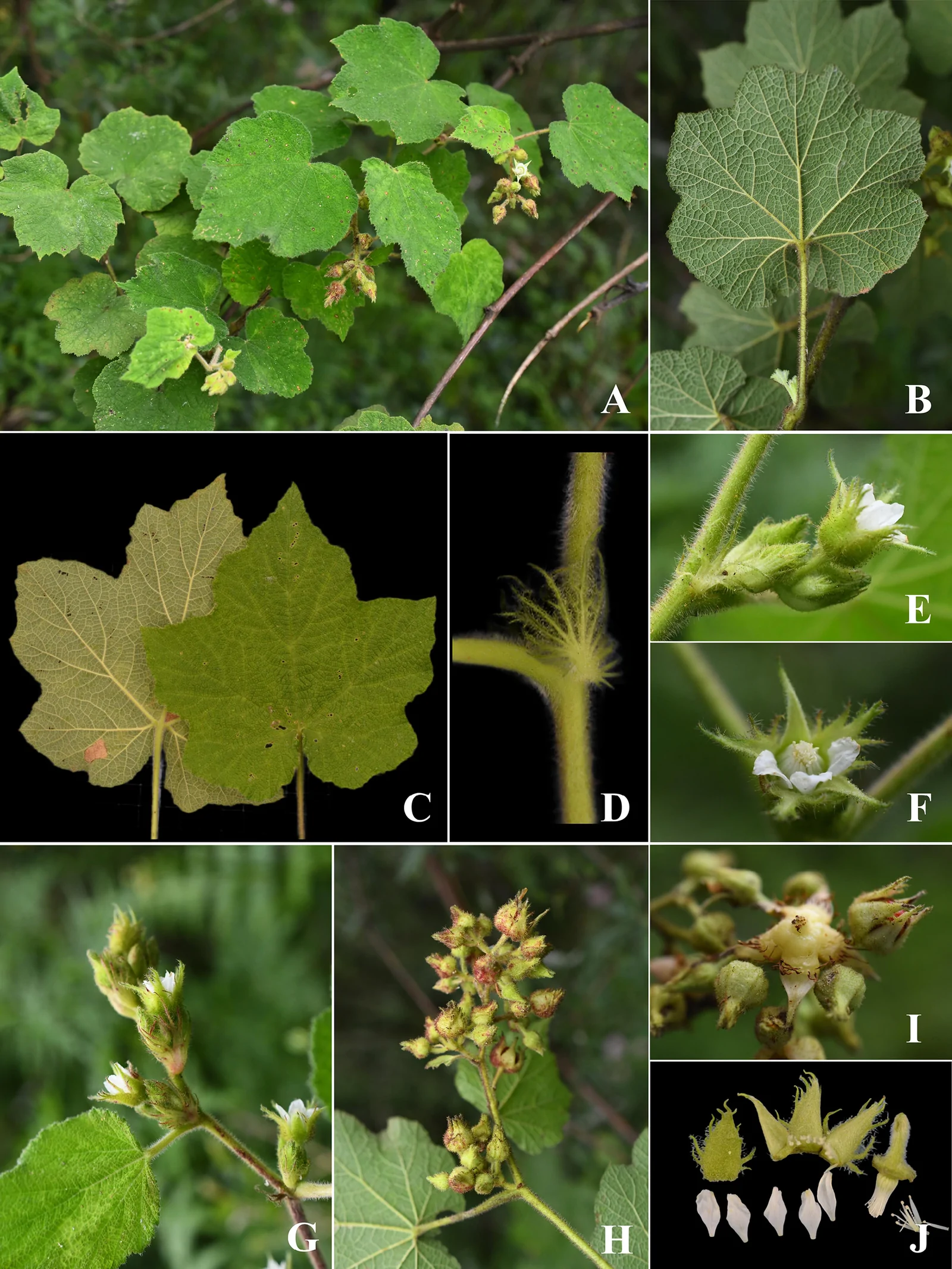
Morphological characteristics of *Rubus phantasmaticus*. **A**. Habit; **B**. Leaf, showing the abaxial surface and subtruncate apex; **C**. Leaf, showing both abaxial and adaxial surfaces and the acuminate apex; **D**. Stipule; **E**. Flower, lateral view; **F**. Flower, top view; **G**. Inflorescence at flowering; **H**. Inflorescence at fruiting; **I**. Receptacle; **J**. Flower, dissected, showing floral anatomy.

**Figure 5.**
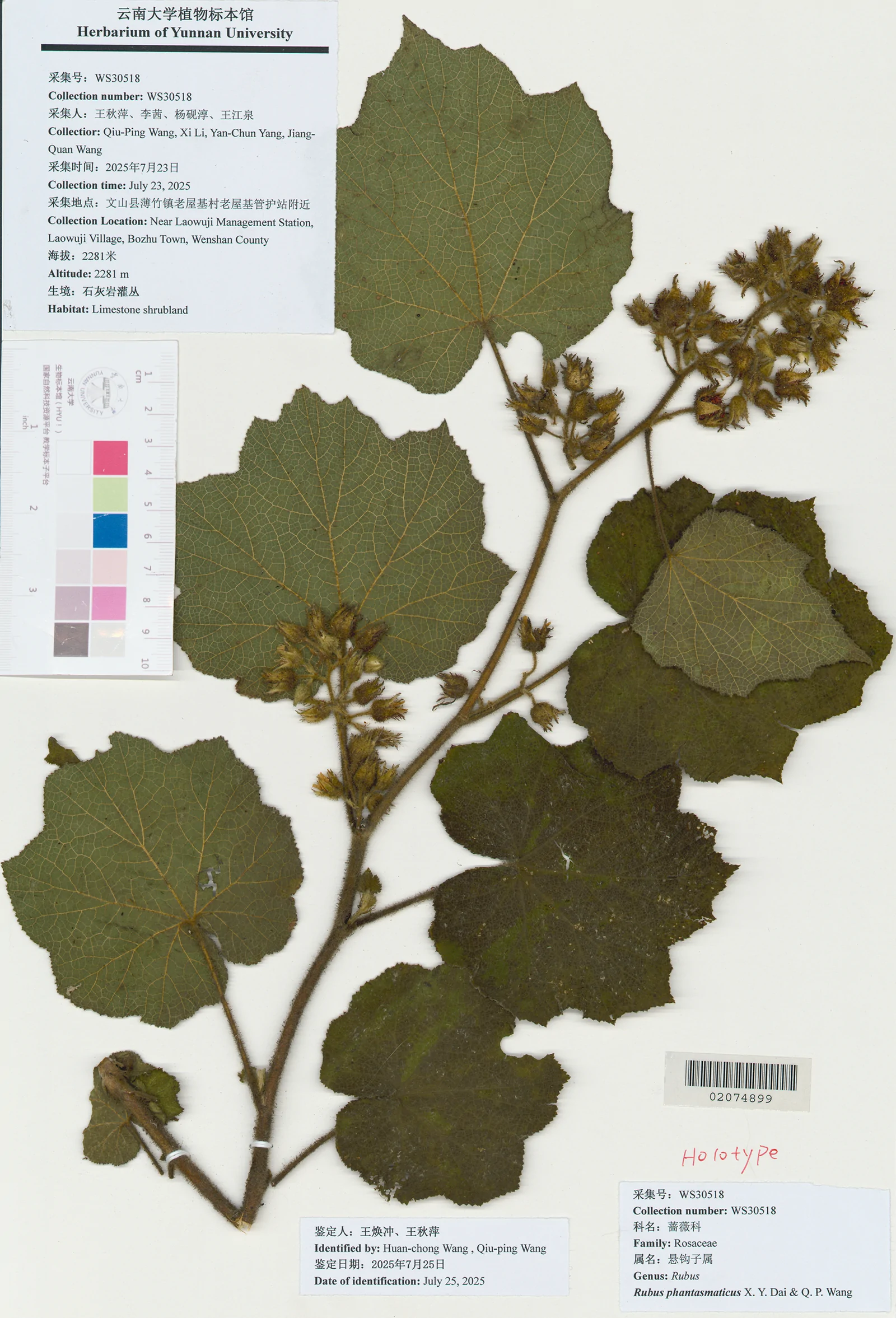
Type specimen of *Rubus phantasmaticus* Q.P.Wang, X.Y.Dai & Huan C.Wang, sp. nov.

##### Diagnosis.

*Rubus phantasmaticus* Q.P.Wang, X.Y.Dai & Huan C.Wang is most closely related to *R. hypopitys*, but differs from the latter by its glandular indumentum throughout the plant (vs. eglandular), arching shrubby habit with stems up to 2 m tall (vs. a dwarf shrub with prostrate to decumbent stems), abaxially villous leaves (vs. densely tomentose when young and becoming sparsely villous at maturity), leaf blade margins usually 5-lobed with acute to triangular lobe apices (vs. shallowly 3–5-lobed with rounded lobe apices), and flowers arranged in narrow panicles or subracemes with three or more flowers (vs. flowers 2–3 or slightly more, arranged in terminal corymbs or solitary in the leaf axils).

##### Description.

Evergreen shrubs, up to 2 m tall. Branchlets terete, reddish brown to brownish, densely villous, intermixed with short glandular hairs, unarmed or sparsely armed with small prickles. Leaves simple; blade cordate to suborbicular, 10–16 × 8–14 cm, apex usually acute to acuminate, rarely subtruncate, base cordate, adaxially pilose, abaxially densely villous, sparsely glandular-hairy along veins, palmately 5-veined at base, lateral veins 1–3 pairs, veins impressed adaxially, slightly rugose or bullate, prominent and reticulate abaxially, margin 5-lobed, rarely 3-lobed, lobes triangular, irregularly coarsely serrate; petiole terete, 4–8 cm long, densely villous and glandular-hairy, sparsely armed with minute prickles; stipules free, broadly obovate, 1.5–1.7 × 1.5–2.0 cm, both surfaces densely villous with intermixed glandular hairs, palmately divided to the base, lobes lanceolate or linear-lanceolate, secondarily divided, caducous. Inflorescences terminal, narrow cymose panicles or subracemes, up to 9 cm long; axillary inflorescences racemose or clustered; rachis, pedicels, and calyx densely villous with intermixed glandular hairs; pedicels 0.6–0.9 cm long; bracts broad, stipule-like, palmately laciniate; flowers 1–1.5 cm in diam.; calyx villous on both surfaces with intermixed short glandular hairs; sepals in two whorls, three in outer whorl and two in inner whorl, triangular, 1–1.2 × 0.5–0.6 cm, apex caudate or long-caudate, inner sepals entire at margin, outer sepals pinnately shallowly lobed, sepals erect in fruit, rarely reflexed; petals white, obovate or oblanceolate, 0.6–0.8 × 0.4–0.5 cm, apex rounded, slightly shorter than sepals; stamens 40–50, white, 0.5–0.6 cm long, filaments broad and flattened at base, glabrous; pistils 40–50, white, glabrous, styles as long as or slightly longer than stamens. Aggregate fruit red at maturity.

##### Etymology.

The specific epithet is derived from the Latinized form of the Greek noun φάντασμα, meaning ‘ghost,’ alluding to the long-overlooked and misidentified nature of this species, which has persisted unrecognized among known taxa much like a ghost among the living.

##### Phenology.

Flowering from July to August, fruiting from September to October.

##### Distribution and habitat.

**Figure 6.**
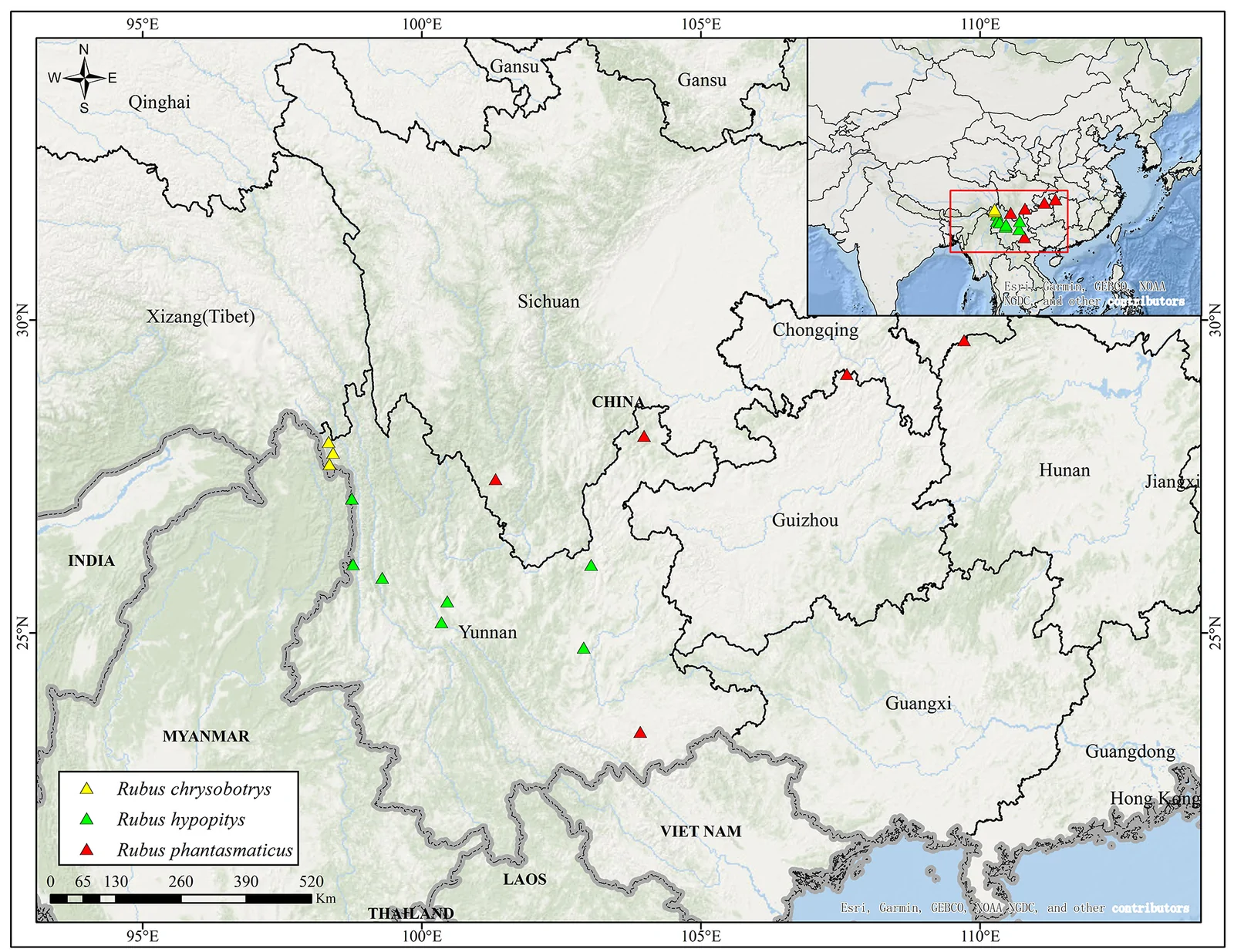
Distribution of *Rubus phantasmaticus* (red triangles), *R. hypopitys* (green triangles), and *R. chrysobotrys* (yellow triangles).

*Rubus phantasmaticus* is endemic to China and occurs in Guizhou, Hubei, Sichuan, and Yunnan (Fig. 6). It usually grows on forest margins along mountain valleys at elevations of 1,000–2,300 m.

##### Additional specimens examined.

China. Guizhou: Zunyi City, Daozhen County, Yangxi Village, Dashahe, 1,000–1,200 m a.s.l., 17 September 2017, *X. Y. Dai 19071105* (YUKU) • Hubei: Xuanen County, Badagongshan, 1,300–1,400 m a.s.l., 25 July 1958, *H. J. Li 5215* (KUN0714954) • Sichuan: Leibo County, Qinghe Village, 24 August 1989, *Q. S. Zhao 1608* (PE01790026) • Yunnan: Zhaotong City, Daguan County, Sanjiangkou, Heilinzi, 1,800 m a.s.l., 28 September 1973, *G. M. Feng et al. 73-78* (KUN0715103).

## Discussion

In this study, molecular phylogenetic analyses of *Rubus phantasmaticus* and its close relatives were conducted based on nuclear ribosomal ITS and complete plastome datasets. The topologies recovered from both datasets consistently resolved *R. phantasmaticus* as nested within R. subg. Malachobatus and most closely related to *R. hypopitys*, a species endemic to southwestern China. Morphologically, *R. phantasmaticus* is characterized by simple leaves, free stipules, and aggregate fruits adnate to the receptacle, all of which are consistent with the diagnostic characters of R. subg. Malachobatus (Focke 1910, 1914; Yu and Lu 1985; Lu and Boufford 2003; Huang et al. 2023). Rubus subg. Malachobatus is morphologically well defined and readily distinguished from other subgenera of the genus. Following the exclusion of R. sec. Lineati and the incorporation of R. subg. Dalibardastrum, it is now recognized as a clearly circumscribed monophyletic group (Alice et al. 2008; Wang et al. 2016; Carter et al. 2019; Huang et al. 2023). Although the circumscription of R. subg. Malachobatus is now relatively well established, its internal classification remains highly problematic. Focke (1910–1914) originally recognized seven sections within R. subg. Malachobatus, but subsequent molecular phylogenetic studies have failed to support this sectional framework. Most of these sections appear to be para- or polyphyletic, and interspecific relationships within R. subg. Malachobatus remain poorly resolved (Alice et al. 2008; Wang et al. 2016; Carter et al. 2019; Huang et al. 2023). Given the incomplete infrageneric classification and poorly resolved sectional relationships, the sectional placement of *R. phantasmaticus* within R. subg. Malachobatus cannot be reliably determined at present.

In the ITS phylogeny, *Rubus phantasmaticus* was most closely related to *R. hypopitys*, *R. lasiotrichos*, and *R. pinnatisepalus*, with these four taxa forming a well-supported monophyletic clade (PP = 0.99, BS = 96). In the plastome phylogeny, *R. phantasmaticus* and *R. hypopitys* were resolved as a moderately supported subclade (PP = 0.85, BS = 80), which was sister to a subclade comprising *R. setchuenensis* and *R. stipulosus* (PP = 1.00, BS = 100). Together, these four species were further grouped with the subclade formed by *R. treutleri* and *R. lyi*, collectively constituting Clade H1 with full support (PP = 1.00, BS = 100). The three species clustered with *R. phantasmaticus* in the ITS tree, together with members of Clade H1 in the plastome tree, are centered in the mountainous regions of southwestern China and share a suite of distinctive morphological traits, including a scandent shrubby habit, palmately veined, suborbicular to broadly ovate leaves, mostly subracemose terminal inflorescences, outer sepals often apically laciniate, and calyces erect in fruit (Yu and Lu 1985; Lu and Boufford 2003).

**Figure 7.**
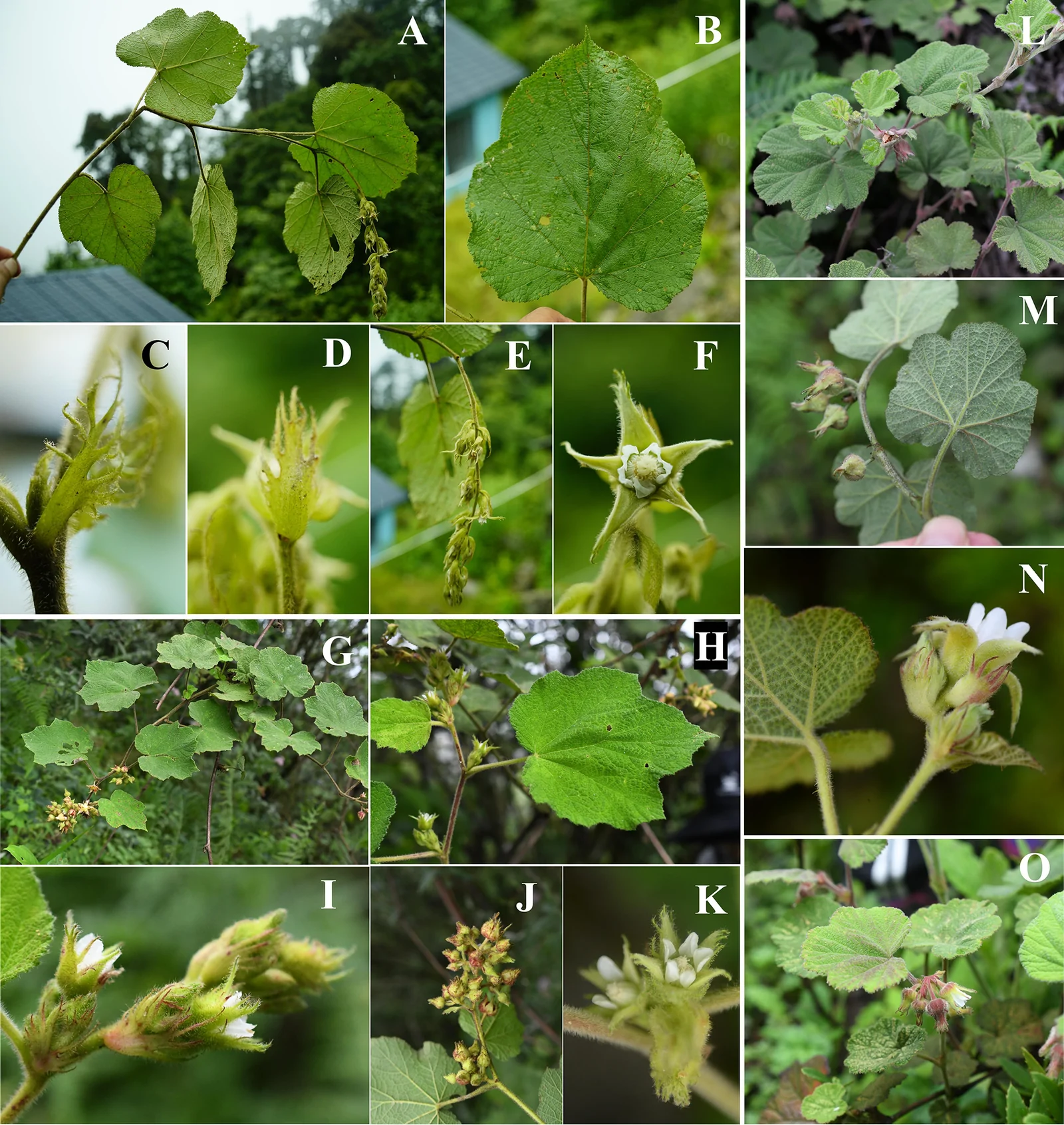
Morphological comparison among *Rubus phantasmaticus*, *R. hypopitys*, and *R. chrysobotrys*. **A–F**. *R. chrysobotrys*; **G–K**. *R. phantasmaticus*; **L–O**. *R. hypopitys*.

Both plastome and ITS phylogenetic analyses indicate that *Rubus phantasmaticus* is most closely related to *R. hypopitys*. The two species are also morphologically similar, sharing cordate to suborbicular leaves with shallowly 3–5-lobed margins, palmately deeply divided stipules, apically laciniate outer sepals, erect fruiting calyces, and petals shorter than the sepals. However, they can be distinguished by several consistent morphological differences (Fig. 7). *R. hypopitys* is a dwarf subshrub with subprostrate stems, whereas *R. phantasmaticus* is a shrub up to 2 m tall. The leaf margins of *R. hypopitys* are shallowly 3–5-lobed with rounded lobe apices, whereas those of *R. phantasmaticus* are usually 3–5-lobed with triangular lobe apices. In addition, *R. hypopitys* bears two or three flowers, or occasionally slightly more, in terminal corymbs or solitary in the leaf axils, whereas *R. phantasmaticus* has narrow cymose panicles or subracemes with no fewer than three flowers. Both species are endemic to the mountainous regions of southwestern China, but their geographic ranges do not overlap (Fig. 6). *Rubus hypopitys* occurs on the Yunnan Plateau and in the southern Hengduan Mountains, whereas *R. phantasmaticus* is mainly found in the transitional zone among the Guizhou Plateau, Yunnan Plateau, and Sichuan Basin. The two species also differ in habitat and elevational range. *Rubus hypopitys* grows in cool, dry montane pine forests and understory thickets at elevations of 2,700–3,000 m (Yu and Lu 1985; Lu and Boufford 2003), whereas *R. phantasmaticus* inhabits warm, humid slopes, forest edges, or thickets at elevations of 1,000–2,300 m.

One specimen of *Rubus phantasmaticus*, Q. S. Zhao 1608 (PE01790026!), was collected from Qinghe Village, Leibo County, Sichuan Province, in 1989. The late L. T. Lu examined this specimen and annotated it on the herbarium label as representing a new variety of *R. chrysobotrys*. This proposed taxon was subsequently validly published by Bi and Lin (2024) as R. chrysobotrys var. glandulosus. However, the phylogenetic analyses showed that *R. phantasmaticus* was resolved in a distinct clade and had no close phylogenetic affinity to *R. chrysobotrys*, contradicting its previous treatment as a variety of that species. Comprehensive morphological comparisons of type material, herbarium specimens, and fresh living plants further showed that the differences between *R. phantasmaticus* and *R. chrysobotrys* extend well beyond the presence or absence of glandular hairs. These differences involve multiple diagnostic characters and demonstrate that the two taxa represent distinct taxonomic entities. In particular, *R. phantasmaticus* can be clearly distinguished from *R. chrysobotrys* by its persistently densely villous abaxial leaf surface (vs. densely tomentose when young, with the tomentum gradually becoming deciduous and leaving only sparse long hairs at maturity), shallowly 5–7-lobed leaf margin with triangular lobe apices (vs. margin undivided or undulate, with rounded lobes), stipules palmately divided to the base with secondarily divided lobes (vs. pinnately divided nearly to the midvein), and abaxial calyx surface pilose and intermixed with glandular hairs (vs. densely yellow-villous and tomentose). Geographically, *R. chrysobotrys* is restricted to the Gaoligong Mountains in northwestern Yunnan (Yu and Lu 1985; Lu and Boufford 2003), a range that is disjunct from that of *R. phantasmaticus* (Fig. 6). Taken together, the morphological, geographic, and phylogenetic evidence supports the recognition of *R. phantasmaticus* as a distinct species rather than a variety of *R. chrysobotrys*. Although elevating Lu’s variety to species rank as *R. glandulosus* would be the most direct nomenclatural treatment, this name was previously used by Bellardi (1793: 230) for a European *Rubus* species and is therefore unavailable for the present taxon.

## Supplementary Material

XML Treatment for Rubus phantasmaticus

## Additional information

### Artificial Intelligence (AI) use

The authors accept full responsibility for the content of the manuscript, including the disclosure of any use of AI.

The following AI tools were used in the preparation of this manuscript:

**AI tools**: ChatGPT.

**Used for**: Language, style and writing.

**Description**: During the preparation of this work, the author(s) used ChatGPT in order to check grammatical errors. After using this tool, the authors reviewed and edited the content as needed and take full responsibility for the content of the publication.

### Author ORCIDs

Q.-P. Wang https://orcid.org/0000-0001-7765-4174

F. Yang https://orcid.org/0000-0003-2076-0130

T.-T. Wang https://orcid.org/0009-0000-1593-1738

H.-C. Wang https://orcid.org/0000-0001-8562-8849

### Data availability

The data supporting the findings of this study are available in the main text and Table 1; newly generated sequences have been deposited in GenBank under the accession numbers listed in Table 1.
